# Continuous, One-pot Synthesis and Post-Synthetic Modification of NanoMOFs Using Droplet Nanoreactors

**DOI:** 10.1038/srep36657

**Published:** 2016-11-08

**Authors:** Sachin R. Jambovane, Satish K. Nune, Ryan T. Kelly, B. Peter McGrail, Zheming Wang, Manjula I. Nandasiri, Shanta Katipamula, Cameron Trader, Herbert T. Schaef

**Affiliations:** 1 Environmental Molecular Sciences Laboratory, Pacific Northwest National Laboratory, Richland, Washington 99354, United States; 2 Energy and Environment Directorate, Pacific Northwest National Laboratory, Richland, Washington 99354, United States; 3 Fundamental Chemical Sciences Directorate, Pacific Northwest National Laboratory, Richland, Washington 99354, United States

## Abstract

Metal-organic frameworks (MOFs); also known as porous coordination polymers (PCP) are a class of porous crystalline materials constructed by connecting metal clusters via organic linkers. The possibility of functionalization leads to virtually infinite MOF designs using generic modular methods. Functionalized MOFs can exhibit interesting physical and chemical properties including accelerated adsorption kinetics and catalysis. Although there are discrete methods to synthesize well-defined nanoscale MOFs, rapid and flexible methods are not available for continuous, one-pot synthesis and post-synthetic modification (functionalization) of MOFs. Here, we show a continuous, scalable nanodroplet-based microfluidic route that not only facilitates the synthesis of MOFs at a nanoscale, but also offers flexibility for direct functionalization with desired functional groups (e.g., -COCH_3_, fluorescein isothiocyanate; FITC). In addition, the presented route of continuous manufacturing of functionalized nanosized MOFs takes significantly less time compared to state-of-the-art batch methods currently available (1 hr *vs.* several days). We envisage our approach to be a breakthrough method for synthesizing complex functionalized nanomaterials (metal, metal oxides, quantum dots and MOFs) that are not accessible by direct batch processing and expand the range of a new class of functionalized MOF-based functional nanomaterials.

Metal–organic frameworks (MOFs) or porous coordination polymers (PCPs) are hybrid porous materials that consist of organic connecting ligands and metal ion-containing nodes or secondary building units (SBUs)^1^,^2^,^3^. In energy applications, MOFs are popular for their structural diversity, tunable pore size, extremely high surface area and hence high adsorption capacities for gas storage and separation, especially CO_2_, H_2_ and CH_4_^4^,^5^,^6^,^7^. Owing to the structural tailorability of organic ligands and the ability to control their assembly with multitopic ligands at the nanoscale, MOFs possess extreme diversity. MOFs and their functionalized analogs often have interesting applications in catalysis^8^, energy storage^9^,^10^, adsorption^11^, efficient contrast agents^12^, nanofluids^13^,^14^,^15^, gas separation^16^,^17^, chemical sensing^16^,^18^,^19^, photoluminescence^20^, light harvesting^21^, biotechnology^22^, and drug delivery^23^. The pure structural form of a MOF, i.e., without any functional groups, may be limited in terms of applications. As such, post-synthetic modification (PSM) is carried out to extend the potential application range of MOFs, by attaching various functional groups to the frameworks. Simply, PSM is a systematic surface functionalization method for the introduction of functional groups in MOFs^24^. If the appropriate functional groups are implanted properly, PSM could also lead to materials that exhibit improved physical and chemical properties^24^. This modification also helps to regulate the overall performance (e.g., colloidal stability at different conditions, self- assembly) and to control its interaction with its environment (e.g., target specific accumulation etc.). Additional advantages of surface functionalization include: (i) preventing aggregation of nanoparticles, (ii) phase transfer; transfer nanoparticles from one solvent (e.g.; organic solvent) to other solvent (e.g.; water), (iii) functionalization with biomolecules enables nanoparticles to interact with specific biomolecules of interest, such as DNA, in biological systems for imaging, delivery and therapeutic use, and (iv) functionalization with fluorescent dyes enables fluorescence labeling of nanoparticles for bio-sensing applications^25^.

To meet the global demand of these interesting classes of MOFs, there is a pressing need for novel synthesis strategies for producing large number of new functionalized nanoparticles and as well large-scale production and development of functionalized nanoparticles with good control over size and morphology. The critical challenge is to control the composition and as well the arrangement of atoms in the nanoparticle system with tunable on production rates. Currently, traditional laboratory approaches such as solvothermal, hydrothermal and microwave methods are used for their synthesis, which suffer from extended reaction times and low quality materials. To control MOF growth and its surfaces at the nanoscale, various surface capping polymers^26^ such as polydadmac^15^ or polyvinylpyrrolidone; PVP have been routinely used^13^,^14^,^15^,^27^. Modulators such as monodentate ligand, which are similar to an organic linker, are also routinely used for modulating MOF growth. However, precise control over size, shape and reduced batch-to-batch variation has led to increasing interest in continuous flow synthesis methods^28^,^29^,^30^,^31^,^32^. Additionally, while PSM is the preferred method for successful functionalization of MOFs, current approaches are extremely time, energy and cost intensive.

In the past decade, a number of methods have been demonstrated for the facile synthesis and PSM of MOFs^33^,^34^,^35^. In direct self-assembly route (Fig. 1, DR), introduction of functional groups onto the ligand could complicate or prevent MOF synthesis due to issues such as steric hindrance, insolubility, thermal instability and competition for SBU coordination^35^,^36^. For functionalized modified ligands, existing reaction condition of the original ligand may not work, making new functionalized MOF synthesis an open-ended synthesis problem^24^,^37^. In addition, the reaction between functional group and SBUs may produce undesired products^37^. For example, the reaction of ZrCl_4_ and 2-acetylamidobenzenedicarboxylic acid (CH_3_CONH−H_2_BDC) in DMF did not produce relevant functionalized MOF product, UIO-66−NHCOCH_3_, instead an amorphous gel was formed^33^,^38^. Therefore PSM (Fig. 1, PSMR) is currently achieved using a process that includes several steps: (a) synthesis of MOFs, (b) removal of starting materials and solvents from the pores retained during synthesis; solvent exchange, (c) drying of MOFs, and (d) functionalization with target functional group. The total time required for these four steps could range from days to few weeks^24^. Moreover, during the MOF synthesis, nucleation takes place at the reaction surface and different nucleation mechanisms arise with the use of large reaction vessel resulting in low quality materials, therefore the size of the reaction vessel becomes a crucial parameter^39^. In addition, the nucleation and growth of colloidal nanoparticles are sensitive to experimental conditions (temperature and concentration), therefore large scale production of nanoMOFs (colloidal sized MOF) with controlled morphology is nearly impossible by simply linearly increasing the volume of the reaction solution in batch synthesis methods.

Currently, conventional and some novel synthetic methods^40^,^41^ yield gram-scale quantities of high quality and defect-free MOFs with uniform size distributions. However, precise control over size and shape and reduced batch-to-batch variation has led to increasing interest in microfluidic synthesis methods^28^,^29^,^30^,^31^,^32^. The automated control, integration, and the potential of in-line quality monitoring are a few of the advantages of microfluidic methods, both continuous phase and droplet based, compared to conventional batch methods^31^. Continuous phase systems are easy to operate but face two drawbacks^31^,^39^,^42^,^43^. First, the tubes in continuous phase may irreversibly foul after extended operation due to direct contact of solvent and starting materials, in addition to MOFs adhering to the inner walls. Second, the viscous drag at the channel walls creates parabolic velocity profile across the tube that causes variation in residence times, which can affect size and property, composition variation of MOFs. The droplet-based systems which are getting increasingly popular for MOF synthesis have overcome the above-mentioned drawbacks and are applied to a wide range of MOFs^29^,^30^,^31^,^39^,^44^,^45^,^46^.

Here, we report a continuous, scalable nanodroplet-based microfluidic route that not only facilitate the synthesis of MOFs at a nanoscale, but also offers the flexibility to directly functionalize them with desired functional groups (e.g., -COCH_3_ and FITC). Our microfluidic continuous route delineated in Fig. 1 combines the above-mentioned four steps required for PSM (Fig. 1-PSMR) into a single step, therefore pushing the field of combined MOF synthesis and its PSM towards industrial setting.

## Preparation of the nanoMOFs and their functionalized analogs

**Figure 2:**
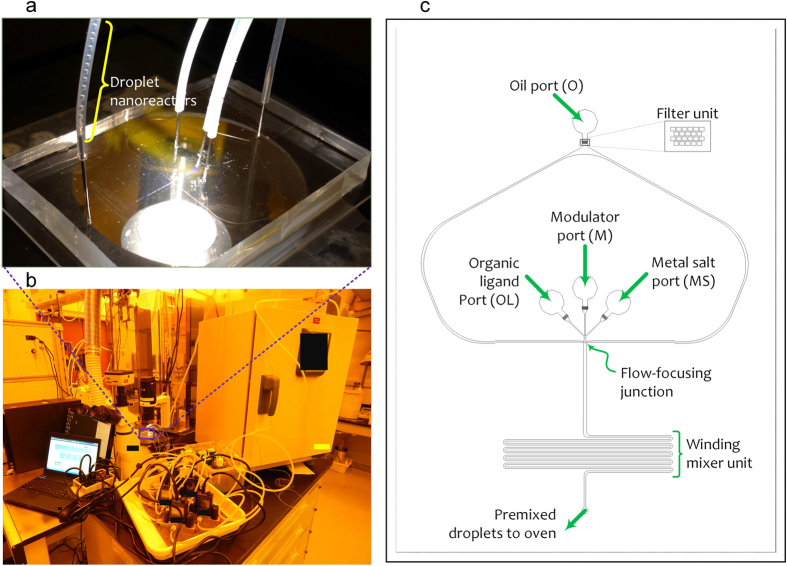
Microfluidic chip for nanoMOF synthesis and post-synthetic modification. (**a**) Droplet based nanoreactors formation using PDMS based microfluidic chip; (**b**) Automated experimental setup to drive microfluidic chip for synthesis and functionalization of nano-sized MOFs and (**c**) Design of microfluidic chip that shows ports and important parts of the chip, flow-focusing junction and winding channel mixer. The chip consists of four ports, feeding oil (O) port, metal salt (MS), organic ligand (OL) and modulator (M) ports and one exit port to transport mixed droplet nanoreactors to a residence tube for subsequent MOF synthesis and PSM inside these nanoreactors.

To address the challenges of conventional synthesis methods, various droplet-based microfluidic devices have been presented during the past few years^29^,^30^,^31^,^39^,^44^,^45^,^46^. These devices offer a number of advantages over conventional batch type reactors. The reduction in droplet volume enables rapid reactions due to extremely small heat capacity and shorter diffusion distances. The ability to conduct a large number of parallel reactions with droplets is another advantage in addition to confinement of reagents with distinct reaction conditions, and isolation of droplets to avoid nonspecific binding of reagents to the channel walls^47^. However, all the existing devices only emphasize on fast and high throughput synthesis of MOFs by using only metal and ligand reagents. Currently reported synthesis methods also seriously suffer from control of size (~200–500 nm) of MOFs due to absence of modulator. The droplet-based microfluidic technique we developed can be exploited as a generic, low-cost, fast, and scalable method for the one-pot synthesis and PSM of MOFs. Our droplet chip works on the principle of flow focusing geometry (Fig. 2c). The device is fabricated using polydimethylsiloxane(PDMS) material and soft lithography technique^48^. The microfluidic chip consists of four ports used for feeding oil (O), metal salt (MS), organic ligand (OL) and modulator (M).

To demonstrate the combined nanoMOF synthesis and PSM, we chose to work with UIO-66-NH_2_ nanoMOF, because the UIO-66-NH_2_ MOF by itself has exceptionally high thermal and chemical stability and offers us an opportunity (due to the presence of amino groups) to incorporate the active functional groups in subsequent PSM reactions^49^,^50^. The continuous MOF synthesis and PSM is achieved in two steps. First, the reagents (MS, OL, M) are injected into oil phase (O) at controlled flow velocities in order to form MOF premixture droplets at the flow-focusing junction where three flows merge. The droplets continue to flow through a winding channel to enhance mixing of reagents. Second, the MOF premixture droplets continue to flow outside the chip through the attached tubing (Fig. 2a, Video-SMV1). The droplets inside the residence tube were allowed to pass through a heating oven (Fig. 2b) kept at 120 °C in order to initiate the nucleation and crystallization of MOFs inside the droplets. The formed MOFs are collected inside the bottle (Video-SMV2) In addition, this method allows us to do real-time monitoring of droplets, which is not achievable in other methods^31^. For real-time online observation, the droplets should be generated in a transparent reactor system or semi-transparent, fabricated from PDMS or polytetrafluoroethylene; PTFE materials, microfluidic systems. In our PDMS chip, we can easily observe the generation and motion of droplets, including mixing of a metal salt solution, organic ligand and modulator reagents, with the naked eye and with the aid of microscope camera. These systems can be microfabricated with a range of materials including PDMS, glass, silicon, stainless steel, ceramics and plastics. However most of the previous systems for MOF synthesis have used assembly of simple tubing’s. The method we developed is highly automated that runs using PDMS based microfluidic chips that in turn are driven by computer controlled and programmable pressure source (Figure S2). Moreover, our method can be scalable by putting multiple microfluidic chips in parallel, like semiconductor processors. PDMS is used for the fabrication of our microfluidic device since multiple chips can be rapidly fabricated by simply curing a premixed PDMS liquid on pre-fabricated silicon master mold. Therefore, the scalability of our approach can be increased by 10 to 20 times using more chips by considering the demand of the production rate *in-situ*. Scalability here refers to the scalability of direct synthesis of functionalized nanosized MOFs (Table 1). Using one microfluidic chip, we can directly synthesize functionalized colloidal nanoMOFs at the flow rate of 5 mL/hour and production rate of 5 mg/hour. The estimated STY production rate is in the range of 0.5 to 1 kg m^−3^ day^−1^ compared to ~1 and 5.8 kg m^−3^ day^−1^ in the small-scale laboratory and microfluidic syntheses as provided in the literature^30^,^51^. Please note that there is no known method for the continuous *in-situ* synthesis and functionalization of colloidal nano sized MOFs. The method presented in this manuscript could be first chip-based platform for the synthesis of MOFs and their functionalized analogs. The production rate at which we produced functionalized MOFs directly in a single step has not been shown by either conventional batch process or by microfluidic-based MOF synthesis until date.

## Characterization of functionalized nanoMOFs

**Figure 3:**
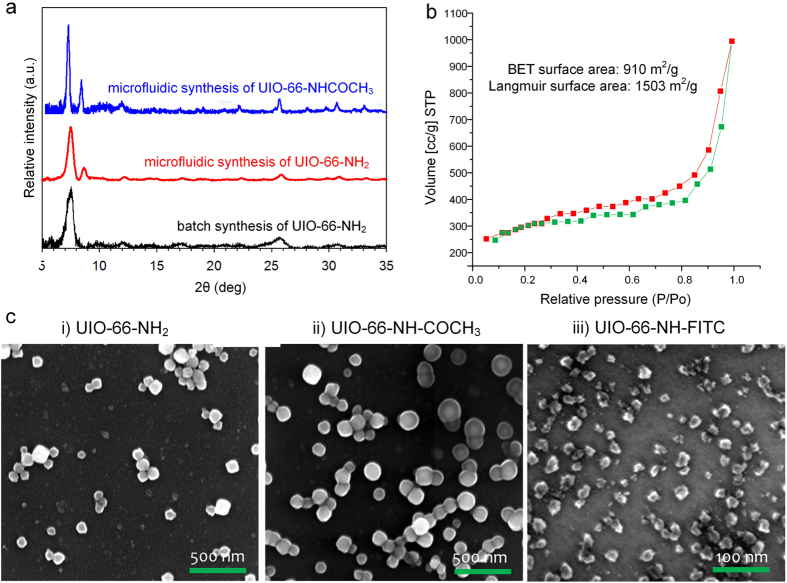
Characterization of nanoMOFs. (**a**) Comparison of PXRD patterns of UIO-66-NH_2_ and its amino analog synthesized through microfluidic device and batch method; (**b**) Nitrogen adsorption isotherms of UIO-66-NH_2_ MOF synthesized using microfluidic system; (**c**) SEM images of (i) UIO-66-NH_2_ (ii) UIO-66-NH-COCH_3_ and (iii) UIO-66-NH-FITC MOFs synthesized from the microfluidic system.

To characterize the crystal structure of as synthesized and functionalized UIO-66-NH_2_ analogs, approximately 5 mg each of UIO-66-NH_2_ and its functionalized analogs synthesized through both batch and microfluidic methods was characterized with powder X-ray diffraction (PXRD). The PXRD patterns of the intrinsic UIO-66-NH_2_ and functionalized analog are presented in Fig. 3a for comparison. PXRD results of both UIO-66-NH_2_ and functionalized UIO-66-NH-COCH_3_ have a prominent peak at 2θ angles of 7.3° and other characteristic peaks at 2θ of 8.4°, 25.7°, and 30.6°. Some broadening of the peaks may be due to the nanocrystallinity of batch intrinsic UIO-66-NH_2_ samples. The PXRD patterns of as synthesized and as well functionalized analogs matches. Therefore functionalization of UIO-66-NH_2_ with acetate groups takes place without losing parent UIO-66-NH_2_ crystallinity in a single step. Our observations are in line with the observations made by Garibay *et al*.^52^, who studied the post-synthetic modification of UIO-66-NH_2_ scaffold and concluded that UIO-66-NH_2_ serve as a tunable microporous scaffold. They also studied the effect of functionalization on the BET surface area. Fig. 3c illustrates the high-resolution scanning electron microscopy (SEM) images of batch intrinsic and functionalized UIO-66-NH_2_ analog samples synthesized using our microfluidic nanoreactors droplet chip. These SEM images indicate the small differences in shape, size and surface morphology of intrinsic and functionalized UIO-66-NH_2_ analog particles. The intrinsic UIO-66-NH_2_ sample has irregular shaped particles in the range of 80–100 nm in diameter (Fig. 3c(i)). When UIO-66-NH_2_ is functionalized with COCH_3_, the SEM image (Fig. 3c(ii)) displays nearly spherical particles in the size range of ~100–150 nm. Interestingly, the size and shape of FITC functionalized UIO-66-NH_2_ analog particles changed significantly. Figure 3c (iii) shows smaller particles (20–30 nm in diameter) with increased irregularity in shape for the UIO-66-NH-FITC sample. It is worth noting that the magnification factor of the SEM image of UIO-66-NH-FITC is different from the other images due to smaller particle size. The SEM images clearly indicate that the shape and size of UIO-66-NH_2_ particles changed after functionalization. It is possible that once the UIO-66-NH_2_ surface is functionalized with FITC it appears to influence MOF growth. It is also possible that filling of the UIO-66-NH-COCH_3_ and UIO-66-NH-FITC pores with polar-solvent molecules (methanol) could also lead to structure breathing, modifying the linker–linker distance^49^ and therefore, producing changes in the size of the parent UIO-66-NH_2_ MOF from ~100 nm to ~20–30 nm. However, further study of this interesting phenomenon is beyond the scope of this paper.

**Figure 4:**
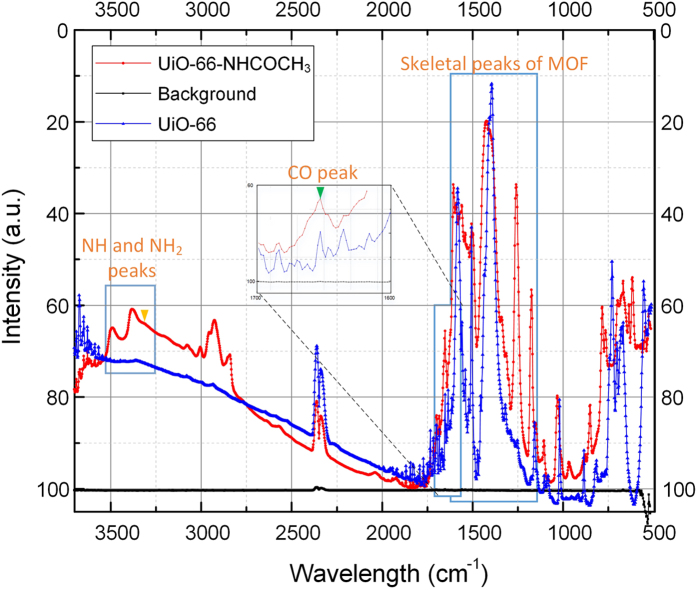
Amino functionalized nanoMOFs. The FT-IR spectrum showing three prominent parts– skeletal peaks related to MOF, CO peaks, NH and NH_2_ peaks of UIO-66- and UIO-66-NHCOCH_3_.

Approximately 10 mg powder of UIO-66-NH_2_ was evacuated under vacuum overnight and used for Brunauer–Emmett–Teller (*BET*) measurements. Nitrogen adsorption isotherms were obtained at 77 K using a multiport volumetric apparatus (Fig. 3b). The BET surface area of our nano UIO-66-NH_2_ is 910 m^2^/g, which compares well with the reported maximum value of 1123 m^2^/g^34^. The detailed area-volume-pore volume summary of UIO-66-NH_2_ is provided in Figure S1. The functionalization reaction between acetic acid and UIO-66-NH_2_ MOF was confirmed by Fourier transform infrared spectroscopy (FT-IR). An FT-IR spectrum was recorded on the sample used for PXRD measurements without any treatment except for evacuating under vacuum for overnight. The summary of FT-IR characteristics wavelengths of post synthetically modified UIO-66-NH-COCH_3_ from conventional batch synthesis^33^ and present nanodroplet-based microfluidic system is provided in Table 2. These wavelengths were also successfully compared with typical wavelength range of characteristics vibrations in the UIO-66-NH-COCH_3_ MOF. The data presented in the Table 2 is a clear evidence of formation of functionalized UIO-66-NH-COCH_3_ MOF using nano-droplet based microfluidic approach. The FTIR spectrum show many skeletal modes for both parent UIO-66 and functionalized UIO-66-NH-COCH_3_ in the region below 1600 cm^−1^ due to vibrations of the organic aromatic ligands (Fig. 4)^33^,^53^. The region below 1600 cm^−1^ is also dominated by key peaks introduced by amino functional group due to post-synthetic modification. More precisely, the UIO-66-NH-COCH_3_ show a strong peak (indicated by small green triangle) at 1652 cm^−1^ in the spectra, a good indication for the presence of the amide ν(C=O) from functional group NHCOCH_3_ (Fig. 4)^33^. As evidence for post-synthetic modification, we also observed erosion of the ν_sy_(NH_2_) and ν_asym_(NH_2_) peaks, at 3495 cm^−1^ and 3380 cm^−1^ respectively. These bands characterize the asymmetrical and symmetrical N–H stretching modes^54^. In addition, corresponding emergence of a new ν(N–H) peak, at 3322 cm^−1^ was also observed as a single weak shoulder peak (indicated by small orange triangle)^33^,^54^,^55^,^56^ i.e. on low-frequency side of the N–H stretching band from overtone of the N–H bending band intensified by Fermi resonance. Such weak shoulder peaks are quite common in PSM literature and they indicate slow progress of PSM^33^. The presence of such peaks also might be coming from the presence of small amount of UIO-66-NH_2_ intermediate formed before the formation of final functionalized analog. Two other strong peaks related to ν(C_ar_-N) are also observed at 1259 and 1340 cm^−1^ 33. The formation of strong peaks at 1257, 1652 and 1340 cm^−1^ clearly indicate progress of post-synthetic modification, however the modification of NH_2_ to NH-COCH_3_ was incomplete after 1 hour of reaction indicating that the longer reaction time might be needed to achieve quantitative modification.

**Figure 5:**
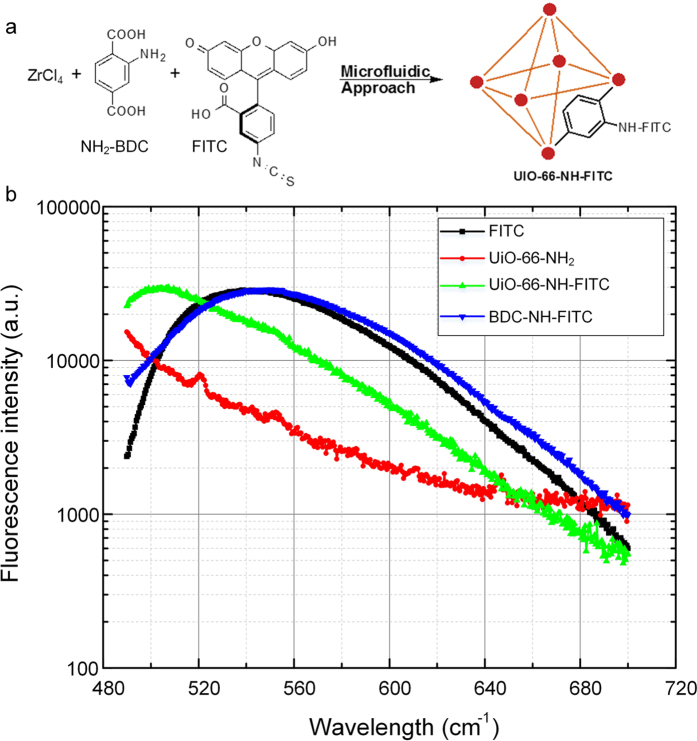
FITC Functionalized nanoMOFs. (**a**) Schematic illustration of FITC functionalization onto UIO-66-NH_2_. (**b**) Fluorescent emission spectra of FITC, FITC tagged to UIO-66-NH_2_ and FITC tagged to linker amino BDC.

Figure 5a illustrate the *in-situ* synthesis and functionalization of FITC onto UIO-66-NH_2_. To investigate the fluorescent behavior of FITC tagged to UiO-66**-**NH_2,_ we excited the suspension of FITC-conjugated analog in methanol at the excitation wavelength of 485 nm and collected the emission spectra band-centered at 530 nm (Fig. 5b). Interestingly, we observed the blue shifted peak at 505 nm^57^,^58^ (green line) for FITC tagged UIO-66-NH_2_ MOF in comparison to expected parent FITC peak at 540 nm (black line). It is also confirmed that UIO-66-NH_2_ MOF has its own fluorescence peak (red line) but at significantly lower wavelength, 430 nm^59^ compared to 505 nm peak of FITC analog. The validity of our blue shift could be confirmed with similar kind of blue shift during the functionalization of FITC to F-UIO^60^ (blue shift to 516 nm) and to UIO-66**-**NH_2_^57^ (blue shift to 516 nm). This shift in the emission spectra could be observed since for cleaning and emission measurement of UIO-66-NH-FITC, we used methanol that has higher polarity index compared to original DMF solvent^49^. Solvent change may not affect intensities significantly since monomer and excimer lifetimes do not show much change with the medium. Therefore, the reason for blue shift could be multifold and so is beyond the scope of this paper. At a higher synthesis and functionalization temperature (120 °C), there is also a potential chance for the formation of an analog formed by bonding FITC directly to amino group on organic linker (2-aminobenzenendicarboxylate, NH_2_-BDC). To validate that we synthesized the pure FITC functionalized MOF analog and not the BDC-NH-FITC analog, we heated the mixture of NH_2_-BDC and FITC under same experimental conditions as that we used in the synthesis of UIO-66-NH-FITC and scanned at same wavelength range for comparison. UIO-66-NH-FITC analog exhibited a peak at 505 nm, which is quite different from pure FITC and BDC-NH-FITC (blue line) that have emission spectra band-centered at 530 nm. Therefore, the detailed study of these fluorescence scans, as illustrated in Fig. 5, unambiguously confirm the formation of FITC functionalized UIO-66-NH_2_ analog.

## Conclusion

In summary, we successfully showed the continuous and scalable nanodroplet-based microfluidic route as a viable platform for the synthesis of MOFs and their functionalized analogs. Confinement of the reactants in nanodroplet resulted in rapid increase in reaction rates, due to enhanced heat and mass transfer. Thus we obtained acetyl and FITC functionalized UIO-66-NH_2_ in a very short time (1 hr) *vs* compared to batch process (6–20 days). Our approach combines four steps currently used in PSM into single step, therefore pushing the field of combined MOF synthesis and its post synthetic modification towards industrial setting. Flexibility of microfluidic system, such as automated control, integration, and the potential of in-line quality monitoring make this approach unique and can be used to access MOF materials that cannot be synthesized otherwise. Although our approach offers a precise control on self-assembly and composition of MOFs at the nanoscale, there remains a challenge on the post synthesis processing such as cleaning and isolation of products and efforts are underway in our laboratory on *in-situ* synthesis and purification.

## Additional Information

**How to cite this article**: Jambovane, S. R. *et al*. Continuous, One-pot Synthesis and Post-Synthetic Modification of NanoMOFs Using Droplet Nanoreactors. *Sci. Rep.* **6**, 36657; doi: 10.1038/srep36657 (2016).

## Supplementary Material

Supplementary Information

Supplementary Video 1

Supplementary Video 2

## Figures and Tables

**Figure 1:**
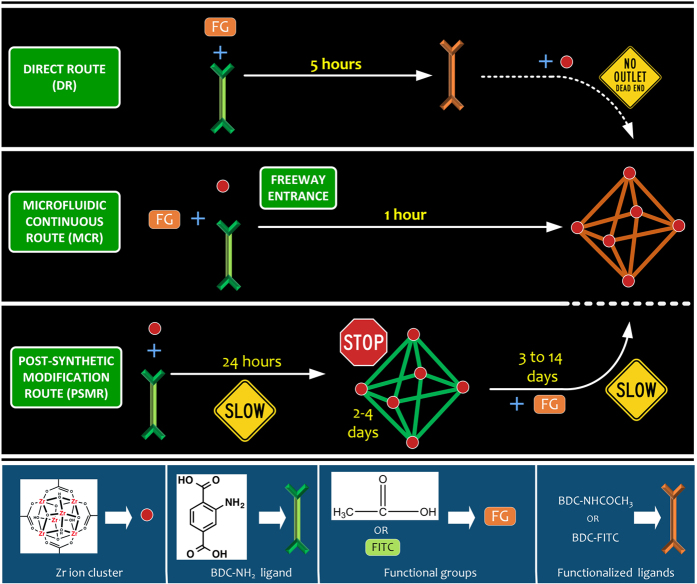
MOF synthesis and post-synthetic modifications *via* different routes. Direct comparison of our microfluidic nanodroplet route with conventional direct and post-synthetic modification of UIO-66-NH_2_.

**Table 1:** Comparison of microfluidic and batch method for combined MOF synthesis and its PSM.

Salient points	Conventional batch	Microfluidics
Automation, control and integration	No	**Yes**
Simultaneous synthesis and post-synthetic modification	No	**Yes**
Scalability	No	**Yes**
Reproducibility	Batch variable	**Yes**
Time required	Days to weeks	**Few hours**
Compositional control	Batch variable	**Possible**

**Table 2:** Comparison of main FTIR characteristic frequencies of post synthetically modified UIO-66-NH-COCH_3_ using present nanodroplet-based microfluidic system and conventional batch PSM.

Wavelength description	Characteristic frequencies (cm^−1^)		
	Typical IR frequency range^61^,^62^,^63^,^64^	Conventional batch PSM^33^	Nanodroplet microfluidic based synthesis + PSM
ν(N-H)	3510–3310	3344	3322
ν_sym_(CH_3_)	2880–2860	2931	2925
Amide I ν(C=O)	1800–1700	1706	1652
Amide II ν(C-N) + δ(CNH)	1575–1480	1544	1541
Amide III δ(NH) + δ(OCN)	1330–1230	1306	1305
ν(C_ar_-N)	1250–1000	1270	1259
