# Supplementary material for: Continuous, One-pot Synthesis and Post-Synthetic Modification of NanoMOFs Using Droplet Nanoreactors
Source: Sci Rep. 2016 Nov 8;6:36657. doi: 10.1038/srep36657 (PMC5099625; doi:10.1038/srep36657)
Supplement: Supplementary Information [file srep36657-s1.doc]

**Supporting Information**

**Continuous, One-pot Synthesis and Post-Synthetic Modification of NanoMOFs Using Droplet Nanoreactors**

Sachin R. Jambovane,^1^ Satish K. Nune,*^,2^ Ryan T. Kelly^1^, B. Peter McGrail^2^ Zheming Wang,^1^ Manjula I. Nandasiri,^1^ Shanta Katipamula,^1^ Cameron Trader,^1^ and Herbert T. Schaef^3^

^1^Environmental Molecular Sciences Laboratory, Pacific Northwest National Laboratory, Richland, Washington 99354, United States

^2^Energy and Environment Directorate, Pacific Northwest National Laboratory, Richland, Washington 99354, United States

^3^Fundamental Chemical Sciences Directorate, Pacific Northwest National Laboratory, Richland, Washington 99354, United States

*Corresponding author: satish.nune@pnnl.gov

Table of Contents

Experimental Section S3

Materials and Methods S3

Microfluidic Device Fabrication S3

Device operation S3

PXRD Measurements S4

SEM measurement S4

FTIR measurement S4

Fluorescence measurement S4

Nitrogen sorption measurements S4

Nanodrop Reactor Experimental Set-up S5

Video-SMV1 on NanoMOFs Synthesis and Functionalization Using Droplet Nanoreactors……..……………………………………………………………………………....S6

Video-SMV2 on NanoMOFs Synthesis and Functionalization Using Droplet Nanoreactors……………………………………………………………………………………..S6

Experimental section

## Materials and Methods

Pure commercial grade chemicals were used for synthesis and post-synthetic modification of UIO-66-NH_2_, UIO-66-NH-COCH_3_, UIO-66-NH-FITC: 1.2 gm Zirconium (IV) chloride (Sigma 221880); 0.93 gm of 2-Aminoterephthalic acid (Sigma 381071); 23.49 gm of 4-Methoxybenzoic acid (Sigma 117390); 2 mL Acetic acid (Sigma A6283), and 1 mg of Fluorescein isothiocyanate isomer I (FITC) (Sigma F7250) and N,N-Dimethylformamide (DMF), anhydrous, 99.8% (Sigma 227056).

**Synthesis of nano UIO-66-NH_2_ (Batch method):** Nano UIO-66-NH2 was synthesized using the reported procedure.^1^ To the fresh vial was added ZrCl_4_ (80 mg, 0.343 mmol), 30 equivalents of modulator 4-methoxy benzoic acid and 30 ml of DMF. The reaction mixture was sonicated for few min to obtain clear solution. An equimolar quantity of amino terephthalic acid (62mg, 0.343 mmol) was added to the reaction mixture solution. The reaction mixture was transferred to the Teflon lined autoclave, sealed it and the reaction mixture was heated at 120 °C for 12 h. The reaction mixture was allowed to cool and pure nanosized UIO-66-NH_2_ was obtained by high-speed centrifugation and repeated washings with ethanol (10 mL) followed by centrifugation.

**Microfluidic device fabrication**: The mask was designed using AutoCAD software (AutoDesk Inc., San Rafael, CA) and printed on glass mask. The mold for fabricating microfluidic PDMS chip was fabricated by a photolithographic technique. At first, the negative photoresist (SU-8 25, Microchem Corp.) was spin-coated onto a 4 in. silicon wafer. This was followed by UV exposure and development. The PDMS chip was fabricated by pouring uncured polydimethylsiloxane (PDMS, GE RTV615; elastomer:cross-linker = 10:1) onto the fluidic mold to achieve a thickness of 5 mm. The poured PDMS on mold was cured for 2 h at 80 °C. The device was peeled off from the mold, and holes were punched for inlet/outlet ports to the flow channels through the thick layer with a 19-gauge punch. Finally, the PDMS chip was placed on a precleaned glass slide (Fisher Scientific Pittsburgh, PA) and kept it in an oven at 80 °C for 24 h to advance curing.

**Device operation**:

To make the channels hydrophobic and oil-repellent, they were treated with RainX (ITW Global Brands, Texas, USA), a commercially available automotive glass treatment, for 2 hours and air-dried at room temperature for 3 hours. Three 100 mL regent bottles were prepared by mixing appropriate amount of dry powders. To prepare the zirconium salt solution (SS), 1.2 gm Zirconium (IV) chloride was dissolved in 100 mL DMF. 0.93 gm of 2-aminoterephthalic acid was mixed in 100 mL of DMF to prepare organic ligand solution (LS). Moderator solution (MS) was prepared by mixing 23.49 gm of 4-methoxybenzoic acid and 1 mg FITC under exclusion of light and moisture. We operated the chip by pneumatic control. To introduce reagents into the flow channels quickly, we applied pressure, which was precisely controlled by using a pressure controllers (Alicat Scientific Inc., Tucson, AZ), to the backside of the reagents. Pressure controllers were connected to digital control box (National Instruments Co., Austin, TX) that was ultimately computer-controlled by a custom-built program. Using pressure controllers, MS, LS, MS and mineral oil were (OS) injected separately into the microfluidic chips, typically at pressures of 36 psi for OS and 35 psi for SS, LS, and MS (Movie S1). After passing the product droplets (Movie S2) through the outlet tubing and oven kept at 120 °C, the product droplets were collected 100 mL bottle. Microscope movies of droplets were taken by using smartphone camera.

**PXRD measurements**

The powdered sample was characterized on a on a D8 Discover XRD unit equipped with a rotating Cu anode (1.54 Å), göbel mirror, 0.5 mm collimator, and 0.5 mm pin hole (Madison, WI).  A GADDS® area detector system positioned at 28.0 °2θ with a measured distance from the sample of 15 cm was used to capture diffraction images.  Collection of individual XRD tracings required 200 seconds with power settings of 45 kV and 200 mA.  Initially, images were processed with Bruker-AXS GADDS® software before importing into JADE® XRD software to obtain peak positions (°2Theta) and intensities.

**SEM Measurement**

SEM imaging was carried out with FEI Helios 600 Nanolab instrument. Images were taken under immersion mode at a working distance of 4 mm and tilt angle of 52°.

**FT-IR measurements**

The FT-IR spectra were recorded on Brucker IFS66 FTIT spectrometer fitted with an IR microscope by placing thin flasks of the solid sample on KBr windows with measurements in transmission mode

**Fluorescence measurement**

The florescence spectra were recorded on a Horiba Fluorolog III spectrometer fitted with 450 W xenon lamp, a single gating monochromator on the excitation side, double monochromator on the emission side and a Hamamatsu R928 photomultiplier tube.

**Nitrogen Sorption measurements**

The surface areas of the sample powders were determined by the Brunauer–Emmett–Teller (BET) method and pore volumes were measured by Barrett–Joyner–Halenda (BJH) method, using nitrogen adsorption/desorption collected with a Quantachrome Autosorb-6B gas sorption system on degassed samples.

|  |
| --- |
| **Figure S1 |** Pore size distribution of UiO-66-NH_2_ nanoMOF synthesized through our microfluidic route. |

**
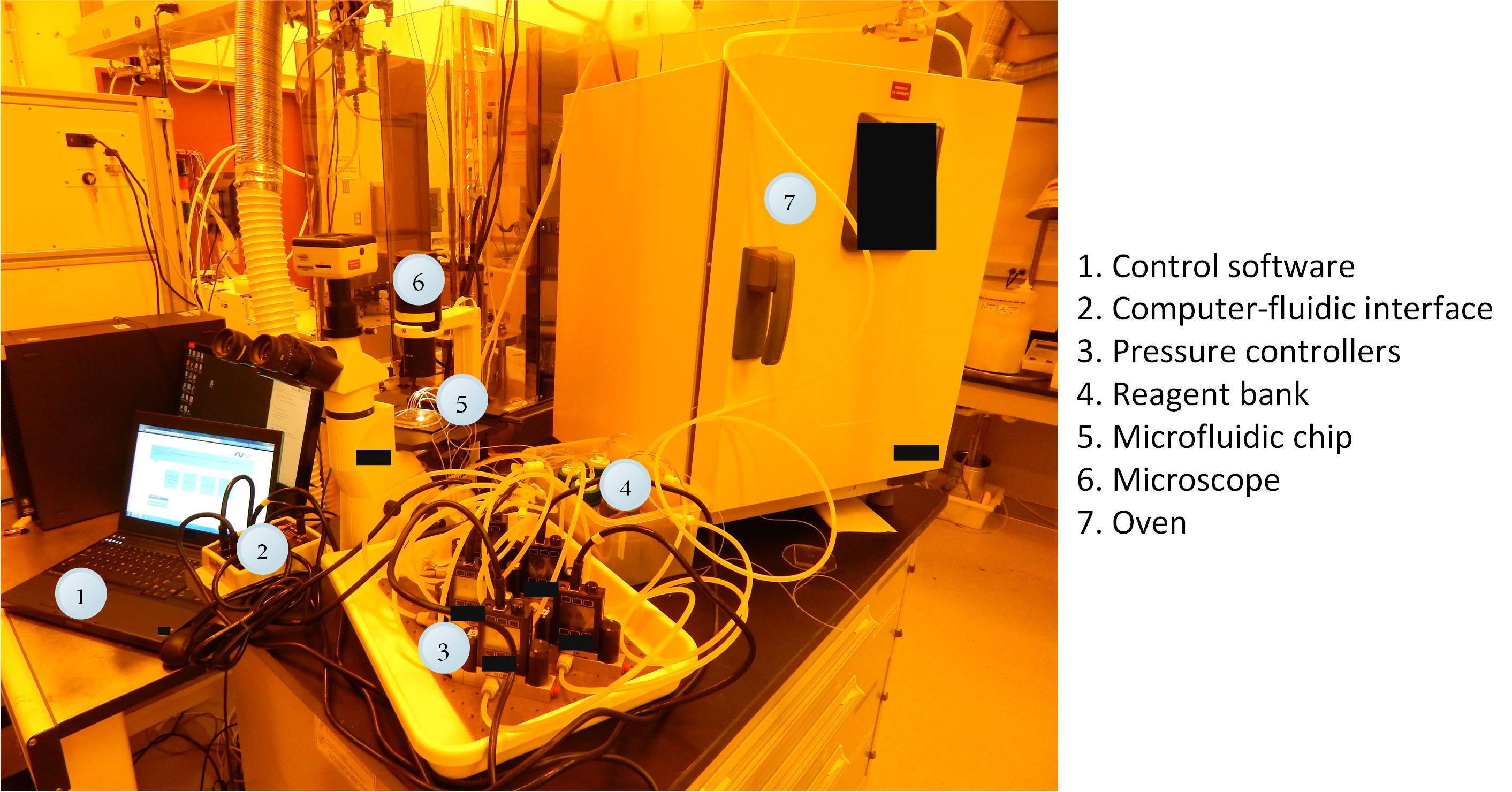
**

**Figure S2 |** Nanodroplet reactor based microfluidic experimental set-up.

**Video-SMV1**-NanoMOFs Synthesis and Functionalization Using Droplet Nanoreactors: Video illustrating the nanodroplets formation using the chip.

**Video-SMV2**-NanoMOFs Synthesis and Functionalization Using Droplet Nanoreactors: Video illustrating the production of MOFs, droplets were retained in the tube throughout.
